# Individual and combined effects of land use and weeds on Cry1Ab/c protein expression and yield of transgenic *cry1Ab/c* rice

**DOI:** 10.1080/21645698.2022.2107385

**Published:** 2022-08-10

**Authors:** Jianmei Fu, Biao Liu

**Affiliations:** a State Environmental Protection Key Laboratory on Biosafety, Research Center for Biodiversity Conservation and Biosafety, Nanjing Institute of Environmental Sciences, Ministry of Ecology and Environment, Nanjing, Jiangsu, China; b Department of Rice Pest, Institute of Plant Protection, Jiangsu Academy of Agricultural Sciences, Nanjing, Jiangsu, China; c College of Life Sciences, Nanjing Agricultural University, Nanjing, Jiangsu, China

**Keywords:** Expression of exogenous protein, fitness, Insect-resistant transgenic rice, uncultivated land soil, weed competition

## Abstract

Considering the anticipated commercial exploitation of insect-resistant transgenic rice and that the planting area of cultivated rice overlaps with wild rice, simulating an escape of transgenic rice from farmlands and exploring its fitness after entering semi-natural or natural ecosystems through uncontrolled seed dispersal or gene flow are critical to understand the resulting potential long-term environmental risks. The expression of foreign Cry1Ab/c protein and vegetative and reproductive fitness of insect-resistant transgenic rice Huahui1 (HH1) and its parental-line Minghui63 (MH63) were studied under four treatments combining land use and weed competition: farmland and uncultivated land under weed control (F-NW and U-NW, respectively), and farmland and uncultivated land without weed control (F-W and U-W, respectively). The expression of Cry1Ab/c was significantly lower in U-NW, F-W, and U-W than that in the control treatment, F-NW. Except for plant height, key vegetative (tiller number and biomass) and reproductive (grain number and grain weight per plant) growth indices of transgenic HH1 were significantly lower than those of the parental-line MH63 in F-NW and U-NW, indicating a significant fitness cost. In F-W and U-W, vegetative growth indices (plant height, tiller number, and biomass) were similar in HH1 and MH63; however, key reproductive indices including seed-set rate were significantly higher in HH1 than in MH63, indicating significant fitness benefits. Although these results support large-scale cultivation of insect-resistant transgenic rice in China, the ecological risk involved is high in farmland or uncultivated land without weed control (F-W and U-W).

## Background

Genetically modified crops have already been planted commercially on a large scale in many countries, bringing important economic and social benefits. However, potential environmental and ecological risks, in addition to biosafety concerns due to the cultivation of genetically modified crops have attracted increasing attention of various stakeholders globally.^1^ Researchers in China, the largest producer and consumer of rice, have conducted extensive research on insect-resistant transgenic Bt (*Bacillus thuringiensis)* rice aiming to reduce the application of chemical pesticides while simultaneously minimizing the impact of insect pests on rice yield.

On October 20, 2009, Huahui1 and Bt-Shanyou 63, two insect-resistant transgenic *cry1Ab/c* rice lines were firstly granted safety certificates by the Ministry of Agriculture of China for production and utilization. Those certificates were renewed in 2020, thereby promoting the commercialization of insect-resistant transgenic rice considerably.^2^ Therefore, under the background of the anticipated commercial exploitation of transgenic rice on a large scale, the study of the fitness of transgenic rice in different semi-natural and natural environments is vital for anticipating the potential risks of its entry into natural ecosystems through different ecological routes, such as uncontrolled seed dispersal or gene flow.

The normal expression of a foreign gene is the biological basis for performing its function but it might influence the fitness of parental crops. In addition, the expression of foreign proteins is closely related to the external environment. Thus, for example, previous studies have demonstrated that, although the level of expression of a foreign protein was significantly lower under stress, such as drought, salinity, or phosphorus deficiency, than under normal conditions, nonetheless it was still expressed.^3–5^ If a foreign protein is expressed normally in natural ecosystems, this may alter the fitness and influence the evolutionary potential of wild parental rice in natural populations. Therefore, investigating the expression of foreign proteins posed by insect-resistant transgenic rice under natural conditions might determine what potential factors are responsible for differential Bt expression.

Investigating the fitness of insect-resistant transgenic rice under natural conditions is also vital in view of its commercial promotion. To date, numerous studies have found that insect-resistant transgenic rice showed superior field performance to the parent rice line under high insect pressure or field conditions without insect control, which is the fitness advantage that a foreign gene confers to insect-resistant transgenic rice over the parental rice lines, and the basis for its development and commercial promotion.^6–9^ Xia et al. (2011) reported that, in the field and under high insect pressure, the yield of insect-resistant transgenic *cry1Ab/c* rice cultivars Huahui 1 and Bt-Shanyou 63, was significantly higher than the yield of the corresponding parent rice lines, Minghui 63 and Shanyou 63, respectively.^2^ Another transgenic line, *bt/cpTI*, also showed resistance against insect damage in advanced-generation hybrid weedy rice and wild rice in the field under higher insect pressure, and offered a shelter that prevented insect feeding on hybrid rice, thus resulting in significantly higher yield than that of the parental line.^10–12^ Conversely, other studies have reported that insect-resistant foreign genes may introduce a fitness cost in parental rice either in the presence or absence of target-insect pressure.^3,13,14^ However, very few studies have explored the fitness of transgenic crops upon entering natural ecosystems through uncontrolled seed dispersal or gene flow and other processes of population dynamics.^3,15,16^ To the best of our knowledge, the fitness of insect-resistant transgenic rice in semi-natural and natural growing conditions (i.e., farmland without weed control, uncultivated land with weed control and uncultivated land without weed control), has not been reported.

The environmental safety concerns over the widespread use of transgenic crops are legitimately based on the potentially broad ecological consequences in time and space. Therefore, a current high-priority challenge is to conduct a comprehensive scientific assessment of all the environmental safety concerns over transgenic crops in the shortest possible time. As China is the center of origin of wild rice, inevitably, vast areas of cultivated rice overlap with the areas of distribution of wild rice. If insect-resistant transgenic rice is to be cultivated commercially on a large scale in the future, and it shows major fitness advantages after escaping into the surrounding natural ecosystems, gene flow might occur between cultivated and wild rice that might pose substantial ecological risks. In addition, as target-insect pressure would be controlled in farmlands and natural ecosystems following commercial cultivation of the insect-resistant transgenic Bt rice, the potential environmental risks associated with a change in fitness in natural ecosystems should be evaluated under low insect-pressure conditions.

Based on the foregoing background, the present study simulated an escape of transgenic rice from farmland under conditions of: farmland under weed control (F-NW, control treatment), uncultivated land under weed control (U-NW, semi-natural individual treatment), farmland without weed control (F-W, semi-natural individual treatment), and uncultivated land without weed control (U-W, natural combination treatment) in a glass greenhouse (i.e., low insect pressure without target-insect), (1) to detect the level of expression of the Cry1Ab/c protein in different plant tissues of HH1 rice under different cultivation condition; and (2) to investigate the vegetative and reproductive growth fitness and explore the possible reasons for any changes in fitness of transgenic *cry1Ab/c* rice under the different experimental growing conditions. The findings of the present study will provide a sound theoretical basis for the evaluation of the long-term environmental risks resulting from extensive cultivation of transgenic *cry1Ab/c* rice.

## Materials and Methods

### Rice

The experimental materials used in the present study were insect-resistant transgenic rice line Huahui-1 (HH1), were granted safety certificates by the Ministry of Agriculture of China for production and utilization, and an elite restorer line parental rice (*Oryza sativa* L.) Minghui 63 (MH63). The HH1 was derived from a T51 transformation event of Minghui-63 containing a fused *Bt cry1Ab/c* transgene, based on a particle gun technology. The *cry1Ab/c* transgene was synthesized from the 1,344 bp *cry1Ab* gene (GeneBank accession no. X54939) and the 486 bp *cry1Ac* gene (GeneBank accession no. Y09787), and driven by the rice actin1 promoter.^6^ Results of Southern hybridization showed that the HH1 rice contained one copy of the transgene.^6^ HH1 showed a high level of expression of the delta-endotoxin, which led to 100% larval mortality.^6^ These two lines were provided by the National Key Laboratory of Crop Genetic Improvement, Wuhan, China.

### Land Use Type and Weeds

We simulated four growing conditions by combining two types of land use with or without weed control. Uncultivated land typical yellow topsoil (0–30 cm) was collected from a natural uncultivated land in Jiangning District, Nanjing, Jiangsu (31°37’-32°07’ N, 118° 28’-119°06’ E). Farmland topsoil (topsoil, 0–30 cm, control) was collected from a paddy field in Liuhe District, Nanjing, Jiangsu (32°11’-32°27’ N, 118°34’-119°03’ E). The physicochemical properties of the two soils were determined; organic matter, total nitrogen (N), total phosphorus (P), available P, total potassium (K), and available K were significantly lower in uncultivated land soil than in farmland soil (P < .01, Table S1). According to the Classification of Early Arable Land in Red and Yellow Soil in the Southern Mountains and Hills in the People’s Republic of China, Agricultural Industry Standard NY/T309-1996, the fertility level of the test uncultivated land soil is 8–9, which satisfies the experimental requirement for the uncultivated land low concentrations of key nutrient elements that characterize uncultivated land soils.

Considering the high weed cover in wild uncultivated lands, weed seeds were sown to simulate about 100% weed cover. Selected weeds included barnyard grass (Echinochloa crusgalli L.), sedge grass (Cyperus rotundus L.), weedy rice (Oryza sativa L.), and sprangletop (Leptochloa chinensis L.), all of which grow in farmlands and uncultivated lands around Nanjing city. Seeds of the selected weeds were mixed randomly based on weight at a ratio of 1:1:1:1, and then uniformly sown in each test pot (840 mm length × 560 mm width × 360 mm height) with the same amount of seeds. Meanwhile, the rice seeds were simultaneously and directly sown into the above-mentioned pots at a density of 200 mm × 200 mm along with weed seeds. After 40 d of weed growth the density and the average cover of the weeds were calculated before transplanting rice. The average densities of barnyard grass, sedge grass, weed rice, and Chinese sprangletop per pot were approximately 500 ± 50 plants/m^2^ (dominant weeds), 100 ± 25 plants/m^2^, 50 ± 10 plants/m^2^ and 400 ± 50 plant/m^2^ in farmland without weed control, respectively; and were approximately 455 ± 50 plants/m^2^ (dominant weeds), 120 ± 40 plants/m^2^, 58 ± 12 plants/m^2^ and 390 ± 60 plant/m^2^ in uncultivated land without weed control, respectively, initial weed cover was 100%.

### Experimental Design

A pot experiment was conducted from May to October 2016 in a glass greenhouse at the Nanjing Institute of Environmental Science of the Ministry of Environmental Protection. The site is surrounded by residential and office buildings but no farmland. No rice, vegetables, or any other crops were planted within a 5 km radius around the site, which ensured that the experiment was carried out in an environment free of target-insect pressure.

We simulated four treatments combining land use and weed competition: farmland under weed control (F-NW control treatment), uncultivated land under weed control (U-NW, semi-natural individual treatment), farmland without weed control (F-W, semi-natural individual treatment), and uncultivated land without weed control (U-W, natural combination treatment). Under these experimental conditions, we investigated the fitness of foreign *cry1Ab/c* in rice. Considering the fact that rice seedlings will germinate along with weed seeds under wild uncultivated land, rice seeds were indirectly sown to the large experimental pots (840 mm length × 560 mm width × 360 mm height) with or without weeds. To increase the emergence rate, eight seeds were simultaneously sown in each hole, other seedlings were pulled out to ensure one plant in each hole after emergence. A complete randomized block design was used with 12 plants distributed evenly in each pot with plant spacing at 19–21 cm. Each treatment comprised 10 replicates and spacing between pots was 60 cm. Although heteromorphic sedge exhibited high density at the seedling, tillering, and heading stages, they basically disappeared at grain filling stage, while other weeds emerged over the whole plant life cycle. Overall, weed cover first increased, reaching and remaining stationary at 100% before grain filling, and then decreased to 80% at rice maturity. In the F-NW and U-NW treatments, manual weeding was performed over the entire growing season. No insecticide was applied under any of the four experimental treatments, and all remaining test materials were incinerated and inactivated after the experiment was finalized.

### Investigation of Target Insect Pressure

Parental rice MH63 without the insect-resistance gene was considered respondent of insect pressure index. Investigations were conducted at the jointing and heading stages where the most severe occurrence of rice borers was observed in the field, including dry heart rate (including white ear) caused by borer, and leaf roll rate caused by leaf borer.

### The Expression of Foreign Cry1Ab/c Protein Was Determined by ELISA

On July 20 (tillering), August 5 (jointing), September 5 (heading), September 25 (grain filling) and October 25 (grain maturity), rice stems and leaves were frozen in liquid nitrogen including, 5 HH1 rice plants randomly collected from each pot and mixed into one sample, with 5 biological replicates which were stored at −80°C. Foreign Cry1Ab/c protein was quantified using the QualiPlate Kit for Cry1Ab/c (EnviroLogix Inc., Portland, ME, USA). The detailed procedure was carried out according to Fu et al. (2018).^3^

### Determination of Fitness Components of Vegetative and Reproductive Growth of Rice

Four rice plants were randomly selected from each of 5 pots at tillering, heading, grain filling and grain maturity, to measure plant height and tiller number. Selected plants were marked to ensure that the same individuals were measured over the duration of the experiment. Next, 20 plants were randomly selected (evenly distributed in each pot) to measure the SPAD value on rice leaves with a portable SPAD-502 chlorophyll meter (Konica Minolta Co., Japan) at heading, filling and maturing stages. SPAD values were used as a proxy of total chlorophyll content. Rice plants were randomly selected at maturity and then cut off at ground level and dried at 80°C to constant weight, which was measured on a digital balance (PB602-N, Mettler Toledo) to determine aboveground dry biomass.

To estimate the reproductive growth abilities at the maturing stage of HH1 rice and MH63 rice, the following indices were determined according to Fu et al. (2018).^3^ (1) effective number of panicles per plant; (2) panicle length; (3) panicle weight; (4) number of filled grains per plant; (5) total number of grains per plant; (6) filled grain weight per plant; (7) 1000-grain weight; and (8) seed setting rate. In all treatments, we calculated the average value of plants of the same genotype in each pot, and six pots were randomly selected.

### Data Collection and Analysis

The expression of foreign Cry1Ab/c protein in the same plant tissues and growth stages were compared and analyzed using the independent variable *t*-test under different growing conditions. Spatiotemporal dynamic changes of expression of foreign Cry1Ab/c protein in different plant tissues and at different growth stages were analyzed by Duncan´s multiple comparison test under the same growing conditions.

According to the methods of Burke et al.^17^ and Song et al.,^18^ the independent sample *t*-test was used to test whether the fitness value (HH1 *vs* MH63) was significantly different from “1.00.” The fitness components were grouped according to characteristics associated with the two important lifehistory stages, i.e. vegetative growth and reproduction, and the relative fitness related to each lifehistory stage was calculated as the mean of the relative fitness of all characteristics within this stage. Composite fitness across the whole life-history was the mean of the fitness estimates of the two stages above-mentioned. All the aforementioned statistical analyses were computed by SPSS v.16.0 for Windows (IBM Corp., Armonk, NY, USA).

## Results

There were only a few non-target insects due to the absence of pesticides; these included spiders (Arachnida), ladybugs (Coccinellidae), and locusts (*Locusta migratoria manilensis*) under the four treatment combinations. Further, we did not observe “dead heart,” leaf rolling caused by rice stem borers (*Scirpophaga incertulas, Chilo suppressalis*, or *Sesamia inferens*) or rice leaf borers (*Cnaphalocrocis medinalis*) of *Bt* transgenic rice under the greenhouse conditions. The results indicated that our pot experiments experienced free of target-insect pressure.

### Expression of Exogenous Cry1Ab/c Protein

**Figure 1.**
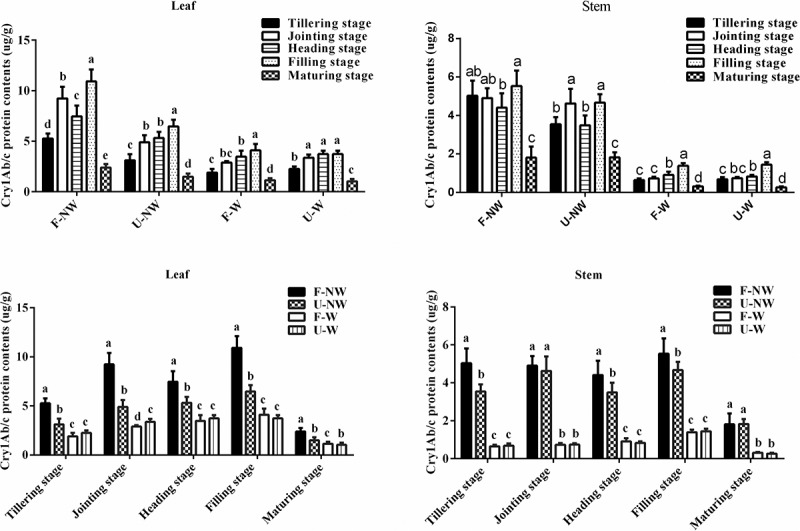
The expression of Cry1Ab/c protein in transgenic rice HH1 leaves and stems grown in four simulated growth conditions at five stages. Lowercase indicated significant differences among the five growth stages grown in the same conditions or grown in four conditions at the same growth stage of HH1 rice according to Duncan’s multiple range test (P < .05).

According to Figure 1, the expression of Cry1Ab/c protein in transgenic HH1 rice leaves and stems exhibited spatiotemporal dynamic changes during different growth stages under the same growing conditions. Cry1Ab/c protein expression first increased and then decreased along the growth stages, with the highest expression observed at the filling stage, and a subsequent significant decrease at the maturing stage.

Under different growth conditions, Cry1Ab/c protein expression in HH1 leaves and stems exhibited some differences in the same growth stages. For example, the expression levels of exogenous Cry1Ab/c protein in U-NW, F-W, and U-W treatments were significantly lower than in the control treatment, F-NW.

In addition, according to the three-way ANOVA, different growth condition, different growing stage, different tissues, and their interaction significantly influenced Cry1Ab/c protein expression (Table S2).

### Vegetative Growth Indices

#### Plant Height

**Figure 2.**
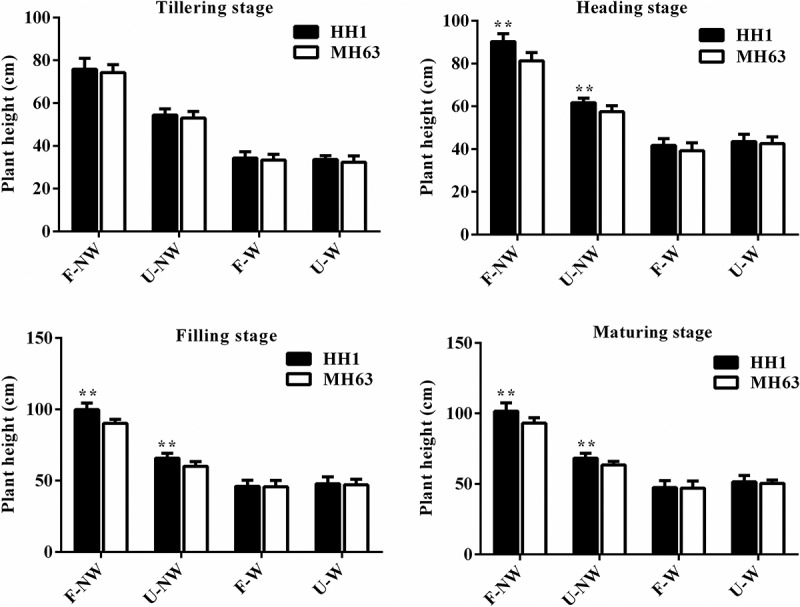
Plant height (Means ± SEM) of HH1 rice and MH63 rice respectively under four growth conditions combining land use and weed competition. Values for HH1 rice with ** are significantly different from those for MH63 according to the *t*-test (P < .01).

Plants of the transgenic-line HH1 and of the parental-line MH63 differed in height at the same growth stage under different growing conditions. Thus, plant height in the transgenic and the parental line in U-NW, F-W, and U-W treatments was significantly lower than in the control treatment, F-NW (P < .01, Figure 2).

The shifting trends in plant height observed in both rice lines during the entire growing period were consistent under the same treatment, both showing a gradual increase concomitantly with plant growth. Except at tillering, HH1 were significantly taller than MH63 by approximately 9–11% at heading, filling, and maturing stages under F-NW conditions (P < .01). Similarly, HH1 plants were significantly taller than MH63 plant by approximately 7–9% (P < .01) at heading, filling, and maturing stages under U-NW individual treatment condition, with the magnitude of the difference in plant height between HH1 and MH63 being lower in U-NW than in F-NW. As for F-W and U-W combination treatments, there were no significant differences in plant height between transgenic HH1 and parental MH63 rice line over the entire growing season.

In addition, according to the three-way ANOVA, the foreign gene, different growing condition, different growth stage, the interaction between the foreign gene and growing conditions, the interaction between growing conditions and growth stage, and the interaction between the foreign gene and growth stages, all influenced rice plant height significantly (Table S3).

### Tiller Number

**Figure 3.**
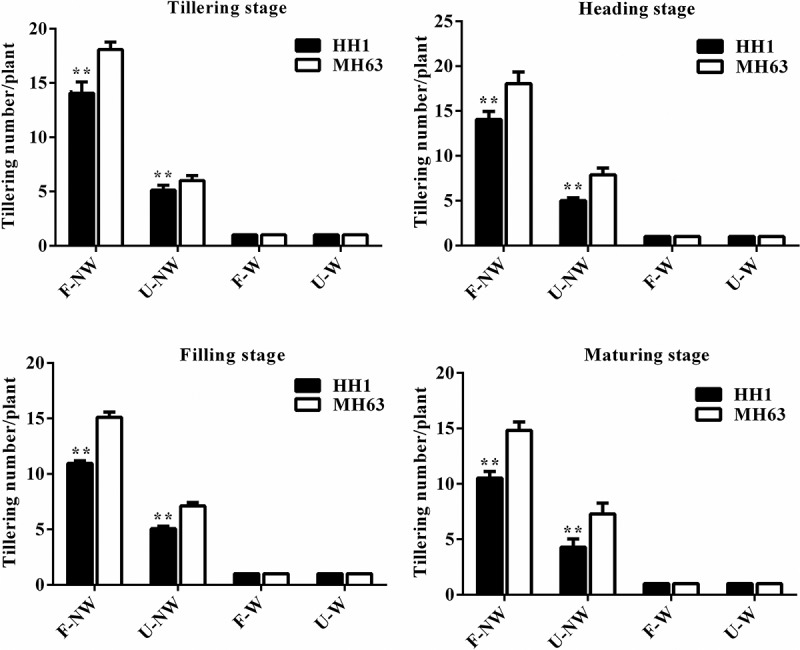
Tiller number per plant (Means ± SEM) of HH1 rice and MH63 rice respectively under four growth conditions combining land use and weed competition. Values for HH1 rice with ** are significantly different from those for MH63 according to the *t*-test (P < .01).

Plants of the transgenic line HH1 and of the parental-line MH63 differed in tiller number at the same growth stage under different growing conditions. Thus, tiller number in the transgenic and the parental line in U-NW, F-W, and U-W treatments was significantly lower than in the control treatment, F-NW (P < .01, Figure 3).

The shifting trends in tiller number observed in both rice lines during the entire growing period were consistent under the same treatment, both showing a gradual increase concomitantly with plant growth. HH1 tiller numbers were significantly lower than MH63 by approximately 19–29% at tillering, heading, filling, and maturing stages under F-NW conditions (P < .01). Similarly, HH1 plants tiller numbers were significantly lower than MH63 plant by approximately 15–41% (P < .01) at tillering, heading, filling, and maturing stages under U-NW individual treatment condition, with the magnitude of the difference in tiller numbers between HH1 and MH63 being higher in U-NW than in F-NW. As for F-W and U-W combination treatments, there were no significant differences in tiller numbers between transgenic HH1 and parental MH63 rice line over the entire growing season (tiller number was “1.00”).

In addition, according to the three-way ANOVA, the foreign gene, different growing condition, different growth stage, the interaction between the foreign gene and growing conditions, the interaction between growing conditions and growth stage, and the interaction between the foreign gene and growth stages, and the interaction exogenous gene, growth conditions and growth stages all significantly influenced rice tiller number (Table S3).

### SPAD Value of Flag Leaves

**Figure 4.**
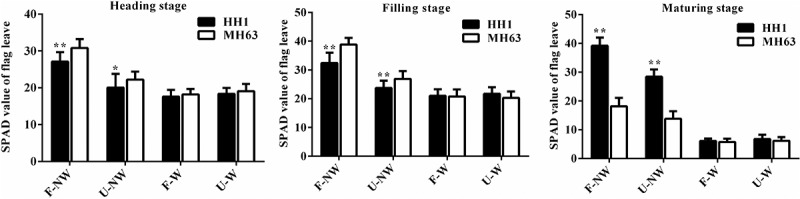
SPAD value (Means ± SEM) of HH1 rice and MH63 rice respectively under four growth conditions combining land use and weed competition. Values for HH1 rice with * and ** are significantly different from those for MH63 according to the *t*-test, respectively (P < .05 or P < .01).

Plants of the transgenic-line HH1 and of the parental-line MH63 differed in SPAD value at the same growth stage under different growing conditions. Thus, SPAD value in the transgenic and the parental line in U-NW, F-W, and W-W treatments was significantly lower than in the control treatment, F-NW (P < .01, Figure 4).

Under F-NW and U-NW conditions, the HH1 rice SPAD values were significantly lower than that of MH63 rice by approximately 12.82% and 10.29% at the heading stage (P < .05 or P < .01), significantly lower than that of MH63 rice by approximately 16.58% and 11.68% at the filling stage, respectively (P < .01), and were significantly higher than MH63 rice at the maturing stage (P < .01). Under F-W and U-W growth conditions, there were no significant differences in SPAD values between the HH1 rice and the MH63 rice at the above-mentioned three stages.

In addition, according to the three-way ANOVA, the foreign gene, different growing condition, different growth stage, the interaction between the foreign gene and growing conditions, the interaction between growing conditions and growth stage, and the interaction between the foreign gene and growth stages, and the interaction exogenous gene, growth conditions and growth stages all significantly influenced rice SPAD value (Table S3).

### Biomass

**Figure 5.**
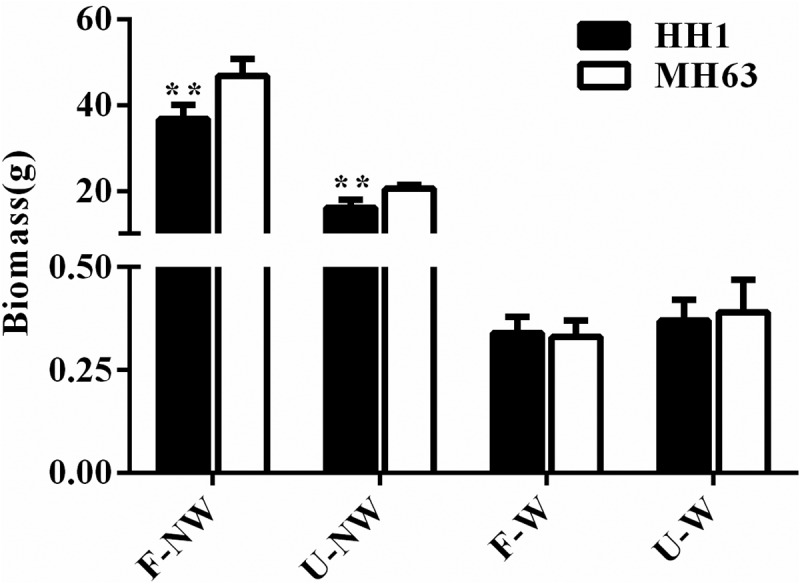
Biomass (Means ± SEM) of HH1 rice and MH63 rice respectively under four growth conditions combining land use and weed competition. Values for HH1 rice with ** are significantly different from those for MH63 according to the *t*-test (P < .01).

Plants of the transgenic-line HH1 and of the parental-line MH63 differed in biomass at the same growth stage under different growing conditions. Thus, biomass in the transgenic and the parental line in U-NW, F-W, and U-W treatments was significantly lower than in the control treatment, F-NW (P < .01, Figure 5).

At maturing stage, HH1 biomass were significantly lower than MH63 by approximately 21% at maturing stage under F-NW conditions (P < .01). Similarly, HH1 plants biomass were significantly lower than MH63 plant by approximately 21% (P < .01) under U-NW individual treatment. As for F-W and U-W combination treatments, there were no significant differences in biomass between transgenic HH1 and parental MH63 rice line.

In addition, according to the three-way ANOVA, the foreign gene, different growing condition and their interaction significantly influenced rice biomass (Table S3).

### Reproductive Growth Indices

Overall, the reproductive indices of HH1 and MH63 showed differences in fitness under different growing conditions and were significantly lower in the U-NW, F-W, and U-W treatments than in the F-NM control treatment (P < .01, Table 1).

**Table 1.** Some reproductive components of HH1 and MH63 rice under four growth conditions combining land use and weed competition.

Reproductive Components	F-NW		U-NW		F-W		U-W	
	HH1	MH63	HH1	MH63	HH1	MH63	HH1	MH63
Effective panicle number/plant	7.67 ± 0.52**	12.50 ± 0.96	3.6 ± 0.49**	5.70 ± 0.45	1.00 ± 0.00	1.00 ± 0.00	1.00 ± 0.00	1.00 ± 0.00
Panicle length	26.17 ± 0.64**	24.23 ± 0.81	22.87 ± 1.16**	20.10 ± 1.44	10.47 ± 0.83*	9.04 ± 1.42	11.48 ± 1.14	11.22 ± 1.75
Panicle weight	27.03 ± 0.97**	33.72 ± 1.93	10.44 ± 1.00**	13.33 ± 0.96	0.22 ± 0.02*	0.20 ± 0.02	0.28 ± 0.04	0.28 ± 0.04
Filled grain number/plant	719.67 ± 57.17**	1063.17 ± 78.77	321.00 ± 35.98*	392.33 ± 37.46	8.22 ± 0.68**	7.14 ± 1.24	12.78 ± 1.68**	9.81 ± 1.69
Filled grain weight/plant	20.65 ± 1.11**	23.42 ± 1.23	8.03 ± 0.90*	9.80 ± 0.93	0.23 ± 0.02**	0.18 ± 0.03	0.32 ± 0.04**	0.24 ± 0.04
Total grain number/plant	927.00 ± 86.36**	1415.00 ± 59.23	347.00 ± 36.16**	433.67 ± 33.95	11.56 ± 1.50	11.33 ± 1.55	14.00 ± 2.26	13.54 ± 2.38
Thousand grain weight	27.88 ± 0.51	26.84 ± 0.41	27.80 ± 0.63	26.84 ± 0.31	25.77 ± 0.67	25.72 ± 0.27	25.89 ± 0.34	25.79 ± 0.77
Seed-setting rate(%)	78.00 ± 5.00	75.09 ± 3.76	92.24 ± 1.13	90.34 ± 0.02	81.73 ± 8.89**	63.93 ± 9.74	91.81 ± 6.16**	72.75 ± 5.37

Means ± SEM followed by HH1 rice with * and ** were significantly different from MH63 rice according to the *t*-test (P < 0.05 and P < 0.01, respectively).

While mean panicle length of HH1 transgenic plants was significantly higher than that of the MH63 parent in F-NW, the effective panicle number, panicle weight, total grain number per plant, filled grain number per plant, and filled grain weight per plant were significantly lower in HH1 than in MH63 by approximately 38.6%, 19.8%, 32.3%, 34.5%, and 11.8%, respectively (P < .01). Similarly, while mean panicle length in HH1 was significantly higher than in MH63 in U-NW, the effective panicle number, panicle weight, total grain number per plant, filled grain number per plant, and filled grain weight per plant were significantly lower in HH1 than in MH63 by approximately 36.8%, 21.7%, 18.2%, 20.0%, and 18.0%, respectively (P < .05 or P < .01). On the other hand, panicle length, panicle weight, filled grain number per plant, and filled grain weight per plant in HH1 rice were significantly higher than in MH63 in F-W by approximately 15.8%, 10.0%, 32.2%, 27.8%, and 27.8%, respectively (P < .05 or P < .01), although there were no significant differences in effective panicle number, total grain number per plant or 1000-grain weight between HH1 and MH63 rice. Lastly, in U-W, filled grain number per plant, filled grain weight per plant, and setting rate were significantly higher in HH1 than in MH63 by 30.3%, 33.3%, and 26.2%, respectively (P < .01), while effective panicle number, panicle length, panicle weight, total grain number per plant, and 1000-grain weight did not differ significantly between HH1 and MH63.

Therefore, according to the results of the two-way ANOVA performed on the data, foreign Bt gene expression, growing conditions, and the interaction between the two had a significant effect on most reproductive indices of transgenic HH1 rice (Table 2).

**Table 2.** The effects of growth conditions and rice lines on reproductive indices by Two-way ANOVA analysis.

Reproductive Indices	Growth Condition (Gc)			Rice Line (Rl)			Growth Condition × Rice Line (Rl)		
	df	F	P	df	F	P	df	F	P
Effective panicle number/plant	3	1,327.08	0.00	1	253.64	0.00	3	95.70	0.00
Panicle length (cm)	3	429.32	0.00	1	17.74	0.00	3	1.87	NS
Panicle weight	3	2,905.60	0.00	1	81.95	0.00	3	36.78	0.00
Grain number/plant	3	2,865.19	0.00	1	207.99	0.00	3	127.42	0.00
Filled grain number/plant	3	1,766.93	0.00	1	111.27	0.00	3	66.82	0.00
Filled grain weight/plant (g)	3	2,955.50	0.00	1	35.98	0.00	3	14.19	0.00
Seed-setting rate (%)	3	19.00	0.00	1	37.02	0.00	3	7.56	0.00

P < 0.05 indicated significant difference; NS indicated no significant difference.

### Fitness

According to Table 3, with regard to vegetative fitness, HH1 rice had a significant fitness cost compared to MH63 rice overall. For example, tiller number, biomass presented significant fitness cost under the F-NW and U-NW conditions. Under the F-W and U-W conditions, there was no significant difference in fitness in vegetative growth between the HH1 and the MH63 rice.

**Table 3.** Fitness for vegetative and reproductive components of HH1 *vs*. MH63 rice under four growth conditions combining land use and weed competition.

Life-History Stage		Variable Measured	F-NW	W-NW	F-W	W-W
Vegetative growth components	Fitness	Height	1.08 ± 0.01*	1.07 ± 0.01*	1.03 ± 0.03	1.03 ± 0.03
		Tiller number	0.76 ± 0.04*	0.70 ± 0.02*	1.00 ± 0.00	1.00 ± 0.00
		Biomass	0.79 ± 0.02*	0.79 ± 0.02*	1.02 ± 0.03	1.01 ± 0.01
	Composite fitness		0.88 ± 0.01*	0.85 ± 0.01*	1.02 ± 0.01	1.01 ± 0.01
Reproductive components	Fitness	Effective panicle number per plant	0.61 ± 0.03*	0.63 ± 0.01*	1.00 ± 0.00	1.00 ± 0.00
		Panicle length (cm)	1.08 ± 0.01*	1.14 ± 0.02*	1.16 ± 0.03*	1.02 ± 0.03
		Panicle weight (g)	0.80 ± 0.03*	0.78 ± 0.02*	1.10 ± 0.02*	1.00 ± 0.02
		Filled grain number per plant	0.68 ± 0.02*	0.82 ± 0.02*	1.15 ± 0.02*	1.30 ± 0.01*
		Filled grain weight per plant (g)	0.88 ± 0.03*	0.82 ± 0.03*	1.28 ± 0.02*	1.33 ± 0.02*
		Grain number per plant	0.66 ± 0.02*	0.80 ± 0.02*	1.04 ± 0.03	1.03 ± 0.03
		Thousand grain weight (g)	1.04 ± 0.03	1.04 ± 0.04	1.00 ± 0.00	1.00 ± 0.00
		Seed-setting rate (%)	1.04 ± 0.03	1.02 ± 0.01	1.28 ± 0.02*	1.26 ± 0.02*
	Composite fitness		0.85 ± 0.02*	0.88 ± 0.00*	1.12 ± 0.00*	1.12 ± 0.00*
The whole life-history	composite fitness		0.86 ± 0.02*	0.87 ± 0.02*	1.07 ± 0.06*	1.06 ± 0.06*

Fitness ratio was defined as agronomic traits of HH1 *vs*. MH63 rice; Composite fitness (Total fitness) was defined by the average value of all fitness for vegetative traits or (or and) reproductive traits. * indicates fitness significantly more than or less than 1.00 according to *t*-test (P < 0.05).

With regard to reproductive fitness, HH1 rice had a significant fitness cost when compared to MH63 rice under the F-NW and the U-NW conditions overall. For example, effective panicle per plant, panicle weight, total grain number per plant, filled grain number per plant, and filled grain weight per plant had significant fitness costs. Under the F-W and U-W conditions, the reproductive growth of HH1 rice presented significant fitness benefits overall. For example, filled grain number per plant, filled grain weight per plant, and seed setting rate presented significant fitness benefits.

Overall, for the estimation of composite fitness across the two lifehistory (Vegetative growth and reproduction), compared with the parent MH63, HH1 rice had a significant fitness cost under the F-NW and the U-NW conditions, or significant fitness benefits under F-W and U-W.

## Discussion

The expected development and commercial exploitation of insect-resistant transgenic rice plants on a large-scale demands a scrupulous assessment for their foreign protein expression, vegetative and reproductive fitness that might escape farming systems and enter natural ecological systems. Here, we evaluated these indices above-mentioned under farmlands and uncultivated lands in the presence or absence of weed competition without target-insect pressure. Seemingly, this may contribute to understand the potential long-term environmental risks when transgenic rice plants enter semi-natural and natural ecosystems in the future.

### Growing Conditions Affect Foreign Cry1Ab/c Protein Expression

The normal expression of insect-resistant foreign genes is the biological basis for providing insect resistance and influencing the fitness of recipient crops. Here, we showed that the expression of exogenous Cry1Ab/c protein in HH1 rice leaves and stems exhibited spatiotemporal dynamic changes over the entire growing cycle under the four growing conditions tested. Our results are consistent with results of spatiotemporal dynamic changes in Bt protein expression in transgenic crops in farmlands.^19–22^ In addition, the expression of foreign Cry1Ab/c protein in HH1 rice leaves and stems was significantly lower under individual and combined stress treatments (U-NW, F-W, and W-W) than under control treatment (F-NW), indicating that stress inhibited the expression of the foreign protein. Jiang et al. (2018) reported that Bt protein expression in three insect-resistant transgenic rice lines (cry1C*, cry2A, and *cry1Ab/c*) decreased significantly by 35.0%, 36.3%, and 37.2%, under drought relative to control conditions, respectively.^5^ Further, Fu et al. (2019) observed that Bt protein expression of transgenic *cry1Ab/c* rice under saline conditions was significantly lower than in farmland.^3^ Other similar studies reported that the expression of the resistance-related foreign protein in insect-resistant transgenic cotton and maize correlated negatively with stress.^23–28^ Overall, although the expressions of foreign insecticidal Bt genes driven by a constitutive promoter, the results reported herein and those previously reported demonstrate that Bt proteins in transgenic crops are affected by environmental stress, plant growth and development, and parental background to some extent.^23,29,30^ A possible explanation is that the expressions of exogenous genes is usually regulated at post-transcription or translation levels and the transgene silencing is environmentally and developmentally regulated. Our study showed that weeds (appear as a new factor) are competitor of transgenic variety HH1 in term of fighting for light, taking nutrient form soil and others, and significantly affecting the growth and development of the HH1, thus affecting the expression of insecticidal Bt in HH1. This was consistent with previous report that the expression of exogenous proteins was significantly correlated with phenotypic differences in crops.^30^ However, the foreign protein can still be expressed, which might influence the vegetative and reproductive growth fitness of transgenic rice in such stressful environments.

### Growing Conditions Affect Vegetative Growth Fitness of Transgenic cry1Ab/c Rice

Vegetative growth fitness refers to the ability of a crop to grow and compete. We showed that plant height, tiller number, SPAD value, and biomass were significantly lower under individual and combined stress treatments U-NW, F-W, and U-W than in F-NW, the control situation in our experiments. This finding was similar to the findings of previous studies reporting that vegetative growth of insect-resistant transgenic rice, cotton and maize were significantly lower under salinity or drought stress than under control conditions.^3,23,27,28^ Except for plant height being significantly higher in HH1 than in MH63, other vegetative growth indices, such as tiller number and biomass were significantly lower in HH1 than in MH63 over most of the plant life cycle in F-NW and U-NW treatments without target-insect pressure, suggesting a significant fitness cost. Similarly, Chen et al. (2006) observed that tiller number was significantly lower in transgenic Bt/CpT1 rice plants than in plants of the parental line under low target-insect pressure on a rooftop^31^; furthermore, Jiang et al. (2013) observed that the biomass of insect-resistant transgenic cry1C* rice was significantly lower than that of the parent line under low natural insect pressure in the field^32^; Fu et al. (2018) reported that the tiller number and biomass of *cry1Ab/c* transgenic rice were significantly lower than those of parental MH63 rice in saline-alkaline soil in the absence of significant target-insect pressure.^3^ In addition, significant fitness cost in terms of plant height, root length, and other vegetative growth indices in other Bt transgenic rice, compared with their parents under natural low insect pressure in the field, have also been reported.^13,14^ Overall, the Bt genes often seems to confer obvious vegetative growth disadvantages to parental rice lines under low insect pressure due to the expressions of them. Conversely, there were no significant differences in the aforementioned vegetative growth indices over most of the life cycle between HH1 and MH63 under F-W or U-W treatments. The most likely explanation for these observations might be that the expression of foreign Cry1Ab/c protein was very low in F-W and U-W treatments, whereby the fitness cost in terms of vegetative growth for HH1 did not show.

### Growing Conditions Affect Reproductive Growth Fitness of Transgenic cry1Ab/c Rice

Non-targeted effects of external Bt genes on plant traits have raised concern in transgenic rice breeding. These non-targeted effects usually cause negative variations in yield components. Reproductive fitness refers to the abilities of a plant to produce offspring and cause environmental risk, which is affected by an energy trade-off between vegetative and reproductive growth under some growing conditions. Here, the reproductive indices of HH1 and MH63 rice under individual and combined stress treatments U-NW, F-W, and U-W were significantly lower than under control treatment, F-NW, including filled grain number and filled grain weight per plant. These results were consistent with findings of previous studies, which reported that the reproductive indices in insect-resistant transgenic cotton and parental cotton under flooding-stress conditions were significantly lower than under normal growth conditions.^27,33^ Additionally, filled grain number and filled grain weight per plant, in HH1 were significantly lower than in MH63 in F-NW and U-NW treatments without target-insect pressure, showing a significant fitness cost that may be closely related to lower tiller number, biomass, and other vegetative growth indices in HH1, compared to MH63 during key developmental stages, in turn causing a greater fitness cost to the reproductive ability of HH1 rice. Chen et al. (2006) and Xia et al. (2010) observed that reproductive indices of transgenic *Bt/CpT1* rice, including grain number per plant, grain weight per plant, and effective panicle number were significantly lower than in the parental-line MH86 under greenhouse and field conditions at low target-insect pressure.^31,34^ In addition, other studies have reported that low total yield due to low seed-set rates of transgenic cry1C* rice and transgenic cry2A* rice was significantly lower than that of parental-line MH63 under natural or low target-insect pressure in the field.^8,35,36^ Significant fitness costs for different reproductive indices, such as grain number per panicle, seed-set rate, total yield, and 1000-grain weight have also been reported for transgenic *cry1Ac* rice, transgenic *cry1Ab* rice, and transgenic *cry1Ab/c* rice under natural low target-insect pressure in the field.^13,14^ Based on our own results and those previously reported, foreign Bt genes usually confer obvious reproductive growth disadvantages to Bt transgenic rice plants under greenhouse or field conditions at low target-insect pressure due to the expressions of them.

Other studies and our previous studies shown that, compared with the parents, even though plants harboring foreign genes may demonstrate relatively low or high composite fitness across the whole life-history, they usually do not perform with uniform inferiority of all characteristics at every life-history stage.^3,17,18^ Here, key reproductive indices in HH1 rice including, filled grain number per plant, filled grain weight per plant, and seed-set rate were significantly higher than in MH63 in F-W and U-W treatments without target-insect pressure, evidencing significant fitness benefits. In addition, the high seed-set rate of HH1 might be a key factor explaining the yield advantage of the transgenic line, indicating that the reproductive and ecological risks associated with HH1 under treatments of F-W and U-W were significantly different from those under treatments of F-NW and U-NW. Fang et al. (2018) found that total grain number in transgenic *EPSPS Arabidopsis thaliana* was significantly higher than in parental *A. thaliana* under high temperature or drought stress without target glyphosate, indicating significant fitness benefits.^37^ A likely explanation for the above results may relate to the expression of foreign Cry1Ab/c protein: including the fitness cost may relate to high Cry1Ab/c protein expression in HH1 in F-NW and U-NW treatments, whereas the fitness benefit may relate to a very low Cry1Ab/c protein expression and to the effect of an energy trade-off between vegetative and reproductive growth in HH1 under treatments of F-W or U-W.^31,38^ Based on these results, from an agricultural production perspective, transgenic HH1 rice showed a high reproductive growth ability in the absence of weed control (under F-W and U-W treatments), and the expression of foreign Cry1Ab/c protein significantly reduced the potential yield loss caused by target insects, which is highly beneficial for the future, large-scale adoption and use of HH1 in China. However, stronger reproductive ability shown by HH1 often contributes to the establishment of its populations in such natural or semi-natural ecosystems with the associated potential ecological risks.

## Conclusions

**Figure 6.**
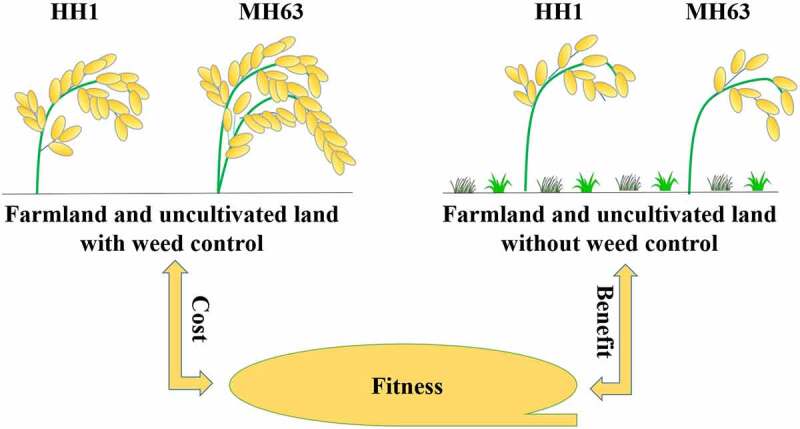
A significant fitness cost of HH1 compared to MH63 under farmland and uncultivated with weed control and a significant fitness benefit between them under farmland and uncultivated land without weed control.

Here, we demonstrated that transgenic *cry1Ab/c* rice-line HH1 showed weaker vegetative and reproductive growth abilities than its parent-line MH63 in farmland or uncultivated land under weed control, which indicated a significant fitness cost. In contrast, transgenic HH1 exhibited an overall stronger reproductive growth ability than parental MH63 rice in farmland or uncultivated land without weed control, which might be conducive to the establishment of transgenic populations and the associated potential ecological risks (Figure 6). Furthermore, we observed that abiotic stress factors, such as uncultivated land soil, and biological factors, such as weed competition, had different effects on the fitness of transgenic HH1 rice. Notably, we did not include the impact of insect pressure in our study design considering that the population of the insect targeting Bt rice in the natural ecosystem is far smaller than that in the farmland ecosystem. If, in the future, Bt rice is to be planted on a large scale, most rice paddy will be under insect pressure; however, Bt rice will not be affected by the attack of these insects, thus promoting the yield. Therefore, further study are much needed to determine the comprehensive effects of weed and target insect pressure on Bt rice, ecological fitness; and to combine weed and target insect pressure jointly analyze the effect of low Bt protein expression on plant insect resistance. Additionally, these findings were recorded during a single-year trial. Consequently, before large-scale commercialization of insect-resistant transgenic HH1 rice is promoted, other natural growth conditions need be simulated for longer periods of time on a case-by-case basis in the future, particularly under natural stress conditions such as saline-alkaline, natural marsh, and weed competition under low target-insect pressure to explore the potential ecological risks of transgenic rice HH1 following large-scale release into natural ecosystems under conditions of low target-insect pressure.

## Supplementary Material

Supplemental Material

## Data Availability

The data sets supporting the conclusions of this article are included within the article and its additional files.

## References

[1] Lu BR, Yang C. Gene flow from genetically modified rice to its wild relatives: assessing potential ecological consequences. Biotechnol Adv. 2009;27(6):1083–91. doi:10.1016/j.biotechadv.2009.05.018. 19463932

[2] Xia H, Lu BR, Xu K, Wang W, Yang X, Yang C, Luo J, Lai FX, Ye WL, Fu Q. Enhanced yield performance of Bt rice under target-insect attacks: implications for field insect management. Transgenic Res. 2011;20:655–64. doi:10.1007/s11248-010-9449-7. 20949317

[3] Fu JM, Song XL, Liu B, Shi Y, Shen WJ, Fang ZX, Zhang L. Fitness cost of transgenic *cry1ab/c* rice under saline-alkaline soil condition. Front Plant Sci. 2018;9:1552. doi:10.3389/fpls.2018.01552. 30405680 PMC6206443

[4] Jiang Y, Ling L, Zhan M, Li CF, Cao CG. Comparison of transgenic Bt rice and its non- Bt counterpart in adaptability to nitrogen deficiency. J Plant Nutr Soil Sc. 2018;181(3):462–70. doi:10.1002/jpln.201700470.

[5] Jiang Y, Ling L, Zhang LL, Wang KX, Li XX, Cai ML, Zhan M, Li CF, Wang JP, Cao CG. Comparison of transgenic Bt rice and their non- Bt counterpart in yield and physiological response to drought stress. Field Crop Res. 2018;217:45–52. doi:10.1016/j.fcr.2017.12.007.

[6] Tu JM, Zhang GA, Datta K, Xu CG, He YQ, Zhang QF, Khush GS, Datta SK. Field performance of transgenic elite commercial hybrid rice expressing *Bacillus thuri-ngiensis* δ-endotoxin. Nat Biotechnol. 2000;18(10):1101–04. doi:10.1038/80310. 11017051

[7] Tang W, Chen H, Xu CG, Li XH, Lin YJ, Zhang QF. Development of insect-resistant transgenic indica rice with a synthetic *cry1C** gene. Mol Breed. 2006;18:1–10. doi:10.1007/s11032-006-9002-9.

[8] Jiang Y, Pan SG, Cai ML, Li CF, Zhan M, Wang J, Mohamed I, Cao CG. Assessment of yield advantages of Bt-MH63 with *cry1C** or *cry2A** genes over MH63 (*Oryza sativa* L.) under different pest control modes. Field Crop Res. 2014;155:153–58. doi:10.1016/j.fcr.2013.09.011.

[9] Jiang Y, Ling L, Zhang LL, Wang KX, Cai ML, Zhan M, Li CF, Wang JP, Chen X, Lin YJ, et al. Transgenic Bt (Cry1Ab/Ac) rice lines with different genetic backgrounds exhibit superior field performance under pesticide-free environment. Field Crop Res. 2016;193:117–22. doi:10.1016/j.fcr.2016.03.014.

[10] Yang X, Li L, Cai XX, Wang F, Su J, Lu BR. Efficacy of insect-resistance *Bt/CpTI* transgenes in F_5_-F_7_ generations of rice crop-weed hybrid progeny: implications for assessing ecological impact of transgene flow. Sci Bull. 2015;60:1563–71. doi:10.1007/s11434-015-0885-x.

[11] Li L, Yang X, Wang L, Yan HX, Su J, Wang F, Lu B-R. Limited ecological risk of insect-resistance transgene flow from cultivated rice to its wild ancestor based on life-cycle fitness assessment. Science Bulletin. 2016;61(18):1440–50. doi:10.1007/s11434-016-1152-5.

[12] Dong SS, Xiao MQ, Ouyang DX, Rong J, Lu BR, Su J, Wang F, Chen JK, Song ZP. Persistence of transgenes in wild rice populations depends on the interaction between genetic background of recipients and environmental conditions. Ann Appl Biol. 2017;171:202–13. doi:10.1111/aab.12365.

[13] Shu Q-Y, Cui H-R, Ye G-Y, Wu D-X, Xia Y-W, Gao M-W, Altosaar I. Agronomic and morphological characterization of *Agrobacterium-transformed* Bt rice plants. Euphytica. 2002;127(3):345–52. doi:10.1023/a:1020358617257.

[14] Kim S, Kim C, Li W, Kim T, Li Y, Zaidi MA, Altosaar I. Inheritance and field performance of transgenic Korean Bt rice lines resistant to rice yellow stem borer. Euphytica. 2008;164(3):829–39. doi:10.1007/s10681-008-9739-9.

[15] Stewart CNJ, All JN, Raymer PL, Ramachandran S. Increased fitness of transgenic insecticidal rapeseed under insect selection pressure. Mol Ecol. 2003;6:773–79. doi:10.1046/j.1365-294X.1997.00239.x.

[16] Su J, Song YN, Yao YX, Chen JM, Wu MJ, Li G. Fitness of transgenic insecticidal rice under different growing condition. Mol Plant Breed. 2013;11:469–76. doi:10.3969/mpb.011.000469.

[17] Song ZP, Lu BR, Wang B, Chen JK. Fitness estimation through performance comparison of F1 hybrids with their parental species *O. rufipogon* and *O. sativa*. Ann. Bot. 2004;93:311–16. doi:10.1093/aob/mch036. PMC4242197 14724120

[18] Burke JM, Carney SE, Arnold ML. Hybrid fitness in the Louisiana irises: analysis of parental and F1 performance. Evolution. 1998;52:37–43. doi:10.1111/j.1558-5646.1998.tb05136.x. 28568157

[19] Olsen KM, Daly JC, Holt HE, Finnegan EJ. Season-long variation in expression of *cry1Ac* gene and efficacy of *Bacillus thuringiensis* toxin in transgenic cotton against *Helicoverpa armigera* (Lepidoptera: noctuidae). J Econ Entomol. 2005;98:1007–17. doi:10.1603/0022-0493-98.3.1007. 16022333

[20] Badea EM, Chelu F, Lacatuşu A. Results regarding the levels of Cry1Ab protein in transgenic corn tissue (MON810) and the fate of Bt protein in three soil types. Rom Biotech Lett. 2010;15:55–62. doi:10.1097/MRM.0b013e3283377af7.

[21] Chen Y, Chen Y, Wen YJ, Zhang X, Chen DH. The effects of the relative humidity on the insecticidal expression level of Bt cotton during bolling period under high temperature. Field Crop Res. 2012;137:141–47. doi:10.1016/j.fcr.2012.08.015.

[22] Wang F, Jian ZP, Nie LX, Cui KH, Peng SB, Lin YJ, Huang JL. Effects of N treatments on the yield advantage of Bt-SY63 over SY63 (*Oryza sativa*) and the concentration of Bt protein. Field Crop Res. 2012;129:39–45. doi:10.1016/j.fcr.2012.01.011.

[23] Jiang L, Duan L, Tian X, Wang B, Zhang H, Zhang M, Li Z. NaCl salinity stress decreased *Bacillus thuringiensis* (Bt) protein content of transgenic Bt cotton (*Gossypium hirsutum* L.) seedlings. Environ Exp Bot. 2006;55:315–20. doi:10.1016/j.envexpbot.2005.01.003.

[24] Luo Z, Dong H, Li W, Ming Z, Zhu Y. Individual and combined effects of salinity and waterlogging on Cry1Ac expression and insecticidal efficacy of Bt cotton. Crop Prot. 2008;27(12):1485–90. doi:10.1016/j.cropro.2008.06.006.

[25] Addison SJ, Rogers DJ. Potential impact of differential production of the cry2Ab and cry1Ac proteins in transgenic cotton in response to cold stress. J Econ Entomol. 2010;103(4):1206–15. doi:10.1603/EC09369. 20857729

[26] Luo JY, Zhang S, Peng J, Zhu XZ, Lv LM, Wang CY, Li CH, Zhou ZG, Cui JJ. Effects of soil salinity on the expression of Bt toxin (Cry1Ac) and the control efficiency of *Helicoverpa armigera* in field-grown transgenic Bt cotton. Plos One. 2017;12:e170379. doi:10.1371/journal.pone.0170379. PMC5242435 28099508

[27] Zhang X, Wang J, Peng S, Li Y, Tian XF, Wang GC, Zhang ZN, Dong ZD, Chen Y, Chen DH. Effects of soil water deficit on insecticidal protein expression in boll shells of transgenic bt cotton and the mechanism. Front Plant Sci. 2017;8:210. doi:10.3389/fpls.2017.02107. 29321788 PMC5732147

[28] Traore SB, Carlson RE, Pilcher CD, Rice ME. Bt and Non-Bt maize growth and development as affected by temperature and drought stress. Agron J. 2000;92(5):1027. doi:10.2134/agronj2000.9251027x.

[29] Adamczyk JJ, Sumerford DV. Potential factors impacting season-long expression of Cry1Ac in 13 commercial varieties of Bollgard® cotton. J Insect Sci. 2001;1:13. doi:10.1673/031.001.1301. 15455073 PMC355897

[30] Rawat P, Singh AK, Ray K, Chaudhary B, Kumar S, Gautam T, Kanoria S, Kaur G, Kumar P, Pental D, et al. Detrimental effect of expression of Bt endotoxin Cry1Ac on in vitro regeneration, in vivo growth and development of tobacco and cotton transgenics. J Biosci. 2011;36(2):363–76. doi:10.1007/s12038-011-9074-5. 21654089

[31] Chen LY, Snow AA, Wang F, Lu BR. Effects of insect-resistance transgenes on fecundity in rice (*Oryza sativa*, Poaceae): a test for underlying costs. Am J Bot. 2006;93:94–101. doi:10.2307/4125475.

[32] Jiang Y, Huang SQ, Cai ML, Li CF, Kong X, Zhang F, Mohamed I, Cao CG. Yield changes of Bt-MH63 with *cry1C** or *cry2A** genes compared with MH63 (*Oryza sativa*) under different nitrogen levels. Field Crop Res. 2013;151:101–06. doi:10.1016/j.fcr.2013.06.017.

[33] Brewer MJ, Odvody GN, Anderson DJ, Remmers JC. A comparison of bt transgene, hybrid background, water stress, and insect stress effects on corn leaf and ear injury and subsequent yield. Environ Entomol. 2014;43(3):828–39. doi:10.1603/EN13309. 24780114

[34] Xia H, Chen LY, Wang F, Lu BR. Yield benefit and underlying cost of insect-resistance transgenic rice: implication in breeding and deploying transgenic crops. Field Crop Res. 2010;118:215–20. doi:10.1016/j.fcr.2010.05.008.

[35] Wang F, Ye C, Zhu LY, Nie LX, Cui KH, Peng SB, Lin YJ, Huang JL. Yield differences between Bt transgenic rice lines and their non-Bt counterparts, and its possible mechanism. Field Crop Res. 2012b;126:8–15. doi:10.1016/j.fcr.2011.09.017.

[36] Ling L, Jiang Y, Meng JJ, Cai LM, Cao GC, Pardha-Saradhi P. Phloem transport capacity of transgenic rice T1c-19 (Cry1C***) under several potassium fertilizer levels. Plos One. 2018;13(3):e195058. doi:10.1371/journal.pone.0195058. PMC5875849 29596474

[37] Fang J, Nan P, Gu Z, Ge X, Feng YQ, Lu BR. Overexpressing exogenous 5-enolpyruvylshikimate-3-phosphate synthase (epsps) genes increases fecundity and auxin content of transgenic *Arabidopsis* plants. Front Plant Sci. 2018;9:233. doi:10.3389/fpls.2018.00233. 29535747 PMC5835131

[38] Liu YB, Ge F, Liang YY, Wu G, Li JS. Characterization of competitive interactions in the coexistence of Bt-transgenic and conventional rice. BMC Biotechnol. 2015;15(1):27–38. doi:10.1186/s12896-015-0141-0. 25928331 PMC4409737

