# Supplementary material for: Individual and combined effects of land use and weeds on Cry1Ab/c protein expression and yield of transgenic cry1Ab/c rice
Source: GM Crops Food. 2022 Aug 10;13(1):156–70. doi: 10.1080/21645698.2022.2107385 (PMC9367653; doi:10.1080/21645698.2022.2107385)
Supplement: Supplemental Material [file KGMC_A_2107385_SM6613.docx]

**Supplementary information**

**Table S1**

Physical and chemical properties of two soils

| Physicochemical property of soil | Farmland | Uncultivated land |
| --- | --- | --- |
| Organic matter (g/kg) | 46.87±1.99^*^ | 11.29±1.33 |
| Total nitrogen content (g/kg) | 2.50±0.46^*^ | 0.74±0.05 |
| Total phosphorus content (g/kg) | 1.66±0.06^*^ | 0.50±0.01 |
| Total potassium content (g/kg) | 12.85±0.04^*^ | 9.86 ± 0.35 |
| Available phosphorus content (mg/kg) | 170.15±11.21^*^ | 16.43±1.93 |
| Available potassium content (mg/kg) | 0.48±0.02^*^ | 0.11±0.01 |

Values for Farmland with * are significantly different from those for uncultivated land according to the *t*-test (P < 0.05).

**Table S2**

The effects of growth conditions, growth stages, tissues, and interactions among them on Cry1Ab/c protein expression in transgenic HH1 rice by Three-way ANOVA analysis

| Three-way ANOVA | Cry1Ab/c protein（μg·g^-1^ FW） | | |
| --- | --- | --- | --- |
|  | df | F | *p* |
| Growth condition (Gc) | 3 | 515.29 | 0.00 |
| Growth stage (Gs) | 4 | 201.99 | 0.00 |
| Tissue (T) | 1 | 473.76 | 0.00 |
| Growth condition × Growth stage | 12 | 17.34 | 0.00 |
| Growth condition × Tissue | 3 | 27.18 | 0.00 |
| Growth Stage × Tissue | 4 | 39.74 | 0.00 |
| Growth condition × Growth stage × Tissue | 12 | 5.37 | 0.00 |

P < 0.05 indicates significant difference; NS indicates no significant difference

**Table S3**

The effects of growth conditions, rice lines, growth stages and the interactions among them on vegetative growth indices by Three-way ANOVA analysis

|  | Plant height (cm) | | | Tiller number | | | SPAD value of flag leave | | | Biomass (g) | | |
| --- | --- | --- | --- | --- | --- | --- | --- | --- | --- | --- | --- | --- |
|  | df | F | P | df | F | P | df | F | P | df | F | P |
| Gc | 3 | 3,289.26 | 0.00 | 3 | 1,531.78 | 0.00 | 3 | 861.83 | 0.00 | 3 | 756.44 | 0.00 |
| Rl | 1 | 74.32 | 0.00 | 1 | 65.78 | 0.00 | 1 | 71.48 | 0.00 | 1 | 25.69 | 0.00 |
| Gs | 3 | 347.88 | 0.00 | 3 | 3.82 | 0.00 | 2 | 578.49 | 0.00 | - | - | - |
| Gc×Rl | 3 | 14.75 | 0.00 | 3 | 41.68 | 0.00 | 3 | 20.50 | 0.00 | 3 | 11.09 | 0.00 |
| Gc × Gs | 9 | 8.34 | 0.00 | 9 | 11.62 | 0.00 | 6 | 66.23 | 0.00 | - | - | - |
| Rl× Gs | 3 | 2.78 | 0.04 | 3 | 1.57 | NS | 2 | 202.15 | 0.00 | - | - | - |
| Gc×Rl× Gs | 9 | 1.43 | NS | 9 | 1.36 | NS | 6 | 76.05 | 0.00 | - | - | - |

Gc: Growth condition, Rl: Rice lines, Gs: Growth stages. P < 0.05 indicates significant difference; NS indicates no significant difference
